# Appearance Over Health: A Phenomenological Study of Obese Adolescents With a Family History of Type 2 Diabetes Mellitus in a Collectivistic Indonesian Context

**DOI:** 10.1002/jgf2.70190

**Published:** 2026-10-22

**Authors:** Anika Candrasari, Vina Yanti Susanti, Oryzati Hilman, Mora Claramita

**Affiliations:** ^1^ Department of Family Medicine and Public Health, Faculty of Medicine Universitas Muhammadiyah Surakarta Surakarta Central Java Indonesia; ^2^ Department of Internal Medicine, Faculty of Medicine, Public Health, and Nursing Universitas Gadjah Mada Yogyakarta Indonesia; ^3^ Department of Family Medicine and Public Health, Faculty of Medicine and Health Sciences Universitas Muhammadiyah Yogyakarta Yogyakarta Indonesia; ^4^ Department of Medical Education, Department of Family and Community Medicine, Faculty of Medicine, Public Health, and Nursing Universitas Gadjah Mada Yogyakarta Indonesia

**Keywords:** adolescent, diabetes, obesity, parental involvement, peer support, prevention

## Abstract

**Background:**

Obesity is a major modifiable risk factor for Type 2 Diabetes Mellitus (T2DM), and Indonesia ranks fifth in the prevalence of diabetes worldwide, coupled with the increasing rate of youth obesity. This phenomenological study explored adolescent obesity as a high‐risk group within Indonesia's collectivistic cultural context to generate qualitative insights that may inform culturally grounded, family‐ and peer‐supported interventions for T2DM prevention. The study does not evaluate the effectiveness of any specific intervention.

**Methods:**

Conducted in Surakarta, Central Java, Indonesia (March 2021–April 2022), the study recruited 11 adolescents, 11 parents, and 9 peers from four primary care clinics using purposive sampling until data saturation. Data were gathered through in‐depth interviews and analyzed using Colaizzi's method.

**Results:**

The majority of adolescents reported being more concerned about their appearance than about their health. The findings also indicated that all groups had limited awareness of T2DM risks. Adolescents perceived intimate relationships as important in shaping healthy lifestyle choices. Peers offered emotional support and encouragement for healthier behaviors, while parents were described as supervising diet and promoting physical exercise.

**Conclusions:**

The findings suggest that health education could potentially address adolescents' appearance‐related concerns as an initial entry point to enhance engagement. For Indonesian adolescents to effectively avoid obesity and lower their risk of T2DM, strategies involving peers and parents may be an important consideration; however, these are presented as implications derived from the qualitative findings, rather than as interventions whose effectiveness has been demonstrated. Future research is needed to empirically test such culturally tailored approaches.

## 1 Introduction

Obesity has emerged as a significant global public health issue that impacts developed as well as developing countries. 17.6% of Indonesian youth were overweight, and 5.4% were obese, according to the 2023 Global School‐based Student Health Survey (GSHS) [1, 2, 3]. Adolescent obesity is associated with a fourfold increased risk of developing T2DM in later life [4, 5, 6, 7]. Lipotoxicity, glucotoxicity, and glucolipotoxicity are among the pathophysiological mechanisms that underlie this risk. These mechanisms contribute to oxidative and metabolic stress, which, in turn, cause pancreatic β‐cell dysfunction and reduced insulin production [8]. The International Diabetes Federation estimates that 4.2 million persons between the ages of 20 and 99 passed away in 2019 due to complications associated with diabetes, underscoring the seriousness of the condition and the significance of early prevention [9, 10]. Thus, there is an urgent need for efficient preventative measures that target high‐risk groups, such as obese adolescents.

Comprehensive diabetes self‐management education that aligns with adolescents' developmental stage and sociocultural environment is crucial, according to the American Diabetes Association (ADA) [2, 11]. People in Indonesia are enmeshed in close‐knit social structures that significantly influence health behaviors, as collectivist values are widely accepted there [12, 13]. Adolescent health outcomes are shaped in this cultural framework by the protective influence of peer and family relationships [14, 15]. Therefore, incorporating the larger family system in health goal‐setting and mutual support should improve Indonesian adolescents' obesity programs [16].

Despite a variety of therapies, there are still some problems with managing adolescent obesity. High dropout rates in long‐term programs have been the subject of several investigations [17], although other studies have revealed no appreciable changes in body mass index (BMI) following treatments [18, 19]. The poor long‐term effectiveness of pharmacological and bariatric therapies further underscores the need for multimodal, culturally sensitive interventions that target behavioral and environmental factors [16].

Most previous studies have concentrated on biomedical or behavioral determinants of obesity, often overlooking adolescents' own perceptions, motivations, and sociocultural realities. Little is known about how Indonesian adolescents conceptualize obesity and the influence of familial and peer relationships within a collectivist society. This knowledge gap limits nurses' and healthcare providers' ability to design contextually relevant, family‐ and peer‐engaged educational interventions that resonate with adolescents' lived experiences. Involving adolescents in decision‐making can lead to more effective, tailored management strategies [16].

This study aims to explore obesity among adolescents in a collectivistic cultural context. By capturing the voices of adolescents themselves, we aim to generate insights that can inform the development of a culturally grounded, family‐ and peer‐supported intervention to prevent T2DM among obese adolescents.

## 2 Methods

### 2.1 Study Settings

This phenomenological research took place from March 2021 to April 2022 in Surakarta, Indonesia's Central Java. Surakarta presents a high prevalence of T2DM and unhealthy eating habits within a collectivist cultural context [20, 21, 22].

### 2.2 Study Design

The lived experiences of adolescents coping with obesity and their social support networks were investigated using a descriptive phenomenological design. Researchers can uncover the complex, nuanced ways people view and interpret obesity within their collectivist cultural context through phenomenological inquiry. This method, which focuses on participants' subjective realities, is ideal for understanding highly personal phenomena such as managing obesity.

### 2.3 Participants and Sampling

Participants included adolescents, their parents, and peers. Adolescents between the ages of 12 and 24 who were overweight or obese (according to WHO BMI‐for‐age standards) and living with a parent were eligible; those with cognitive impairments or severe illnesses were excluded. Parents who had been the primary caregiver for an adolescent, with T2DM for more than 5 years, or who had complications, were eligible; exclusion was based on communication or cognitive impairments.

Peers were close friends aged 12–24 years, nominated by adolescents, with at least 6 months of established friendship; insufficient familiarity led to exclusion. The study employed a deliberate, nonprobability sampling strategy to ensure diversity in body weight, age, gender, and education.

### 2.4 Participant Selection

A multi‐step procedure was used to recruit participants. Based on T2DM risk, medical records from four primary care clinics were examined to find suitable adolescent‐parent pairs. To explain the study, researchers communicated with parents. Adolescents who approved were invited to select their peers.

Informed consent was obtained through printed forms and/or by phone. Recruitment continued until thematic saturation was reached, meaning no new ideas emerged from the interviews. Data collection included 31 participants consisting of 11 adolescents, 11 parents, and 9 adolescent peers.

### 2.5 Data Collection Procedures

A main researcher conducted interviews with two trained assistants to ensure consistency and reflexivity. Some were conducted through video chats or by phone due to COVID‐19 restrictions, but most were conducted in person at clinics or community sites.

Three areas were covered in a semi‐structured guide: (1) difficulties managing weight, (2) experiences with overweight or obesity, and (3) expectations for help from peers and parents. Each session lasted 60–90 min, was audio‐recorded with consent, and transcribed verbatim in Bahasa Indonesia.

### 2.6 Data Analysis

Colaizzi's phenomenological approach, which includes transcript immersion, significant statement extraction, meaning construction, theme clustering, and the creation of a thematic structure, was used to evaluate the data [23, 24, 25, 26]. Participant input and researcher consensus were used to validate the themes.

Despite the data being collected in 2021 and 2022, the analysis considered Surakarta's long‐standing sociocultural background. Several factors have demonstrated remarkable consistency throughout time, including collectivist peer networks, the significant influence of familialism, and traditional eating practices. Emerging themes were cross‐validated with recent research throughout the analytic phase, demonstrating that the cultural values influencing obesity‐related behaviors in this community have not changed and continue to affect health practices.

### 2.7 Trustworthiness

To ensure the rigor and credibility of the qualitative findings, we used multiple strategies to enhance trustworthiness. To gather information from three distinct but connected participant groups—adolescents, parents, and peers—triangulation was used. This approach allowed for a variety of viewpoints on the topic being studied. To assess potential biases, improve interpretative consistency, and ensure alignment with the research aims, we held regular reflexive team talks throughout data collection.

Member verification was achieved by sharing emerging themes with participants, who could verify that the interpretations were correct and consistent with their own experiences. In addition, the data were independently coded by three qualitative analysts. To resolve coding disputes, we did iterative discussions until consensus was reached, thereby reducing subjective bias and increasing analytical reliability.

### 2.8 Ethical Consideration

This work was approved ethically by the Medical and Health Research Ethics Committee of Universitas Gadjah Mada (Reference No. KE/FK/1382/EC/2020). All participants gave their informed consent. Before each interview, participants got thorough information about the objectives of the study, the voluntary nature of participation, and their freedom to leave at any time without facing any repercussions.

Confidentiality and privacy were strictly upheld throughout the research process. All participants were assigned unique codes to anonymize their identities, and any personally identifying information was removed from transcripts and reports. Data were stored securely, with access limited to the core research team.

## 3 Results

Eleven adolescents, 11 parents, and nine peers (31 in total) participated in this study. Most of the adolescent respondents were female (72.73%), with almost the same number of adolescents who were overweight (45.45%) and obese (54.55%) (Table 1. Characteristics of respondents).

**TABLE 1:** Characteristics of respondents.

Adolescents	∑	%	Parents	∑	%	Peers	∑	%
Age (years old)								
16–20	6	54.55	41–45	2	18.18	16–20	5	55.56
21–24	5	45.45	46–50	3	27.27	21–25	4	44.44
			51–55	4	36.37			
			56–60	2	18.18			
Gender								
Man	3	27.27	Man	2	18.18	Man	5	55.56
Woman	8	72.73	Woman	9	81.82	Woman	4	44.44
Level of education								
Secondary (high school)	3	27.27	Secondary (high school)	5	45.45	Secondary (high school)	3	33.33
Tertiary (Diploma‐University)	8	72.73	Tertiary (Diploma‐University)	6	54.55	Tertiary (Diploma‐University)	6	66.67
Weight			Job‐status					
Overweight	5	45.45	Housewives	5	45.45			
Obese	6	54.55	Working	6	54.55			

The results of the interview transcripts, after being analyzed using Colaizzi's approach, provide an example of the process of creating formulated meaning from significant statements (Table 2), an example of how the theme was constructed from different clusters of themes and formulated meanings (Table 3), and the final thematic map (Table 4).

**TABLE 2:** An example of the process of creating formulated meaning from significant statements.

Significant statements	Formulated meaning
“When we go outside, we usually go to the mall and end up eating. If we do sports, we end up eating too.” (Transcript 13, page 9, lines 8–10)	Adolescents, when going out together with friends, often eat. Adolescents, even after exercise, they and their friends also like to eat.
“I've never exercised except when there is a sports schedule at school. At home, I never exercise. From elementary school to middle school, if we were told to run, we would walk, and then when we approached the teacher, we just ran. I want to lose weight, but when I have delicious food, I want to try it.” (Transcript 9, page 6, lines 13–17)	Adolescents never exercise. Adolescents like to try delicious food.

**TABLE 3:** An example of how the third theme, “poor lifestyle” was constructed from different clusters of themes and formulated meanings.

Example of formulated meaning	Theme clusters	Emergent theme
Parents only provide food that children like, with too many carbohydrates and no vegetables	The provision of food by parents with inappropriate nutritional composition	Poor lifestyle
Parents like cooking, so their adolescents like to eat	Parents as wrong role models	
Parents don't like sports, and their adolescents also never play sports		
Adolescents and their peers spend time together (going to the canteen, going to the mall, playing sports)	Bad mutual habits between adolescents and peers	
Adolescents and their peers like fried food, and ice cream		
Adolescents' activities with their peers always end with eating together		
Sedentary adolescent lifestyles	The bad habits of adolescents themselves	
Adolescents like sweet drinks		

**TABLE 4:** The final thematic map.

**First theme**	**Second theme**
*Adolescent Worried about Appearance than Health*	*Adolescent Lack of Health Awareness*
Lack of confidence in appearance Health effects are less of a concern than appearance effects Misperceptions about obesity	Behavior based on surrounding habits Health risks have not yet emerged or are minimal Adolescents lack knowledge and have poor sources of information
**Third theme**	**Fourth theme**
*Poor Lifestyle*	*Parents and Peers Can Support Adolescents Against Obesity*
The provision of food by parents with inappropriate nutritional composition Parents as wrong role models Bad mutual habits between adolescents and peers The bad habits of adolescents themselves	Parents provide support to adolescents Peers provide support to adolescents

### 3.1 Adolescents Worried About Appearance Than Health

Adolescents who are overweight lack confidence in their appearance. Adolescents are more concerned about their appearance than their health.

The first is that obesity is not enough confidence. We want to take photos or hang out with friends, but there is less confidence. The second thing is that it is difficult for us to approach the opposite gender, and there is pressure where fat people cannot fall in love with others (adolescent, 24 years old).

Many friends like this, obese too, but it's no problem (adolescent, 18 years).

People generally have a misperception about obesity. Meanwhile, being thin is also seen as a stigma, as the respondent shared in the following: “*But father likes it when he sees me fat, because being skinny is bad*” (adolescent, 16 years old).

### 3.2 Adolescent Lack of Health Awareness

Adolescent behavior based on surrounding habits (peer): “Still following friends, it's like a friend asking to drink something sweet, so we can't refuse” (adolescent, 21 years old).

Adolescents have not yet felt the impact of obesity on health, or perhaps have only felt it minimally, so it is considered normal, not yet a health threat

No health problems yet (adolescent, 23 years old).

Many friends like this, obese too, but it's no problem (adolescent, 18 years).

The lack of knowledge was evident among adolescents, their parents, and their peers. The information itself was sometimes not known to be correct, primarily because of poor literacy.

I never looked for information about the impact of being overweight on health (adolescent, 16 years old).

It's like Dedy Corbuzier's diet (YouTuber). For example, if we eat lunch about noon and then a few hours after that we can still eat. But after 6 p.m., just drink water (adolescent, 23 years old).

### 3.3 Poor Lifestyle

Our research found that being overweight in adolescents is influenced by many factors in their environment. It can be from their peers, parents, or the habits of adolescents themselves. Parents are role models in the family, and adolescents will imitate their parents' poor lifestyle habits.

…His father's hobby is cooking. So, he likes snacking too (parent, 54 years old).

… including myself, have never exercised (parent, 45 years old).

Together with their peers, adolescents spend most of their time together, so their habits are also mostly the same.

Incidentally, friends from school like to eat (the same food). Usually it's like come on here and buy this (adolescent, 23 years old).

When we go outside, we usually go to the mall and eat. If we exercise together, we end up eating. (peer, 21 years old).

Adolescent lifestyles such as sedentary lifestyles, inappropriate eating, and drinking patterns will increase their weight.

At home, I never exercise (adolescent, 23 years old).

If we go outside, we buy iced tea (adolescent, 18 years old).

### 3.4 Parents and Peers Can Support Adolescents Against Obesity

Adolescents highly expect the involvement of the closest people. In Indonesia, a country whose collectivism is strong, parents contribute to providing support for overweight adolescents.

…for the provision of food at home, the children now eat a lot of vegetables; we arrange it, we prepare extra vegetables (parent, 54 years old).

…you're fat, so it's important for you to exercise, I said that. It can help you lose weight (parent, 51 years old).

Meanwhile, adolescents want their parents to encourage, organize, and prepare appropriate food and drinks, and also invite and accompany them to do sports.

Parents get involved in cooking, reduce fried food, and replace it with stew, then reduce salt (adolescent, 18 years old).

In my opinion, invite sports, rather than sitting or relaxing. It's jogging, not a leisurely walk. (adolescent, 21 years old).

Peer involvement in providing social support is also highly expected because most of their time is spent with them at school and outside the home.

It's encouraging, especially if we're close friends (adolescent, 23 years old).

I want to be supported by everyone. If people around me ask me to eat and drink sweets, it's just useless. I want friends also to invite me to do sports (adolescent, 21 years old).

Peers provide support to adolescents who are overweight by inviting them to engage in sports, keeping friends' feelings positive, and providing the correct information.

I give advice and ask about sports together with us (peer, 21 years old).

It's more about giving advice to lose weight, such as exercising, managing food like snacking, and eating fried foods. In student organizations, we often went up the mountain. Tomorrow, it can be rescheduled to invite him to participate (peer, 21 years old).

### 3.5 Summary of the Findings

This study involved 31 individuals in all, including 9 peers, 11 parents, and 11 adolescents. The majority of adolescent responders (72.73%) were female, while 54.55% were obese and 45.45% were overweight. Through thematic analysis using Colaizzi's methodology, four main themes emerged, reflecting adolescents' opinions, behaviors, and social contexts related to obesity and Type 2 Diabetes Mellitus (T2DM) prevention. These themes are illustrated in Figure 1, which presents the conceptual framework of the study findings.

**FIGURE 1:**
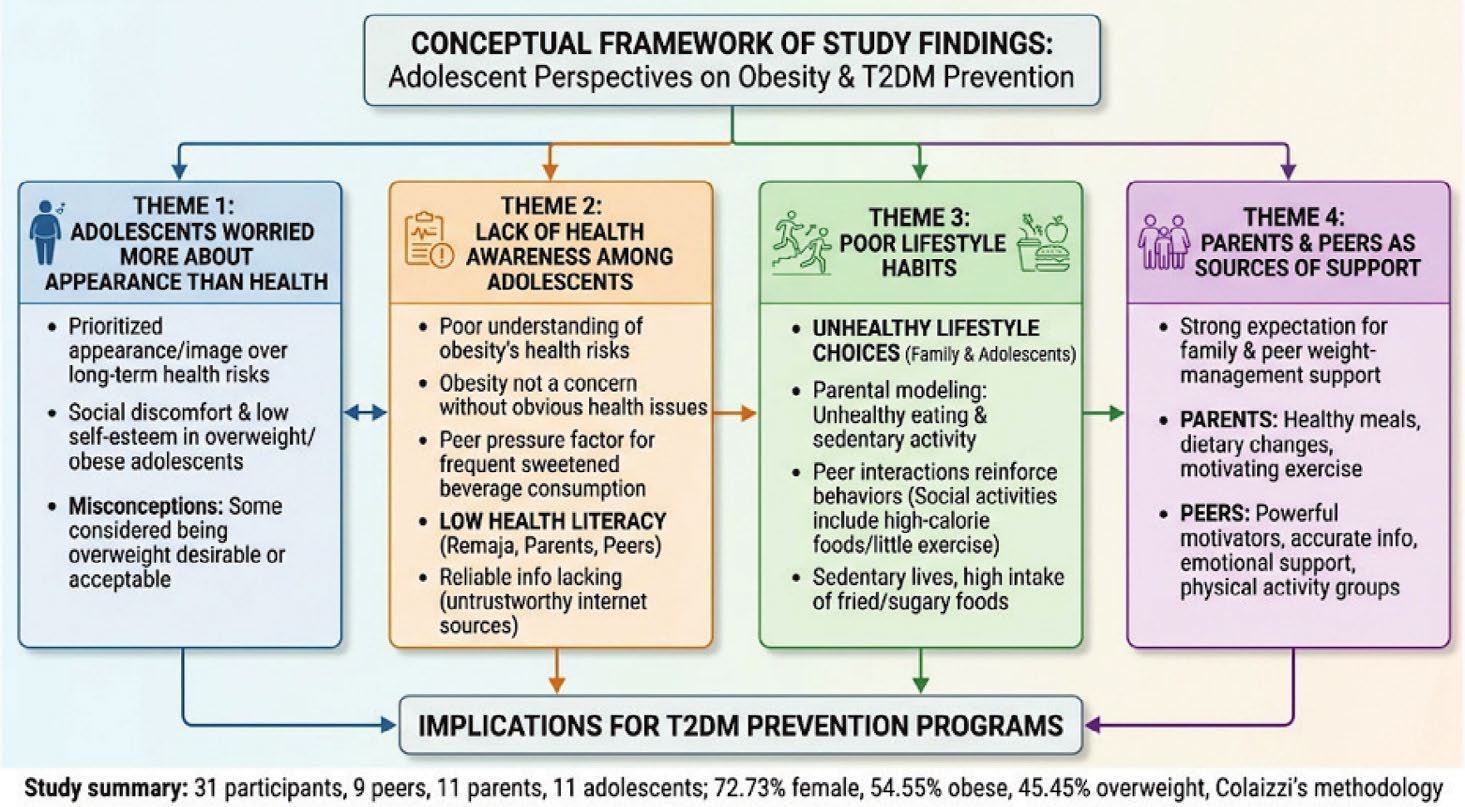
Conceptual Framework of Study Finding (Image generated with Gemini AI).

#### 3.5.1 Theme 1: Adolescents Worried More About Appearance Than Health

Adolescents were more concerned with appearance than with potential health risks. Adolescents who were overweight often expressed social discomfort and low self‐esteem, especially when interacting with their peers. There were many misconceptions regarding body image, with some adolescents considering that being overweight was desirable or even acceptable due to cultural or familial influences.

#### 3.5.2 Theme 2: Lack of Health Awareness Among Adolescents

Adolescents showed a lack of understanding of the health risks associated with obesity. The majority of participants did not view obesity as a concern because they had not encountered any obvious health issues. Peer pressure was a major factor in the development of bad habits like drinking sweetened beverages frequently. The majority of the material regarding diet and weight management came from untrustworthy internet sources, and adolescents, parents, and peers had relatively low levels of health literacy.

#### 3.5.3 Theme 3: Poor Lifestyle Habits

Adolescents and their families exhibited unhealthy lifestyle choices. Adolescents' comparable habits were influenced by their parents' modeling of unhealthy eating and sedentary activity. Since social activities frequently involved consuming high‐calorie foods and little exercise, peer interactions reinforced these behaviors. Respondents frequently reported leading sedentary lives, exercising little, and consuming a lot of fried and sugary foods.

#### 3.5.4 Theme 4: Parents and Peers as Sources of Support

Adolescents stated that they strongly expected peer and family support for weight‐management strategies. Parents offered support by preparing healthier meals, making dietary changes, and motivating their children to exercise. Peers were seen as powerful motivators who might encourage healthy lifestyle choices by providing accurate information, emotional support, and opportunities for group physical activity.

All things considered, these results demonstrate that the complexity of adolescent obesity is in a collectivist cultural setting. To encourage long‐lasting lifestyle modifications and increase adolescents' awareness and motivation to prevent T2DM, family and peer involvement may be a crucial consideration in future strategy development, though its effectiveness has yet to be tested.

## 4 Discussion

The present analysis explores the perspectives of adolescents, parents, and peers regarding the interaction among family, peer, and sociocultural factors in adolescent obesity in Indonesia, particularly the obesogenic environment influenced by dietary behaviors, physical inactivity, midnight snacks, and stress [8, 10, 27]. Despite contributions to health behavior made by individual factors, the findings of this qualitative study highlight the perceived influence of the surrounding environmental context (family and peers) on adolescents' health‐related behaviors [14, 15]. These findings are presented as participants' perspectives and are not intended to demonstrate the effectiveness of any specific intervention.

Family is a microcontext for the development of obesity risk in adolescents [28]. Parental influence, especially in early childhood, has been identified in the literature as an important factor in shaping eating behavior and body image. In this study, interviews revealed that some adolescents were culturally encouraged to gain weight from an early age, and that pressure to eat was commonly reported by participants. These results reflect the perspectives and experiences of the participants and are consistent with earlier research that has examined these parental feeding practices and a higher likelihood of overweight and of involvement in unhealthy weight‐control behaviors [29, 30]. Cultural norms and parenting practices also affect adolescent health‐related behaviors. Positive and supportive parent–child relationships were found to be protective factors for unhealthy lifestyle habits [14, 15, 31]. Parents are not only the child's main caregivers but also role models whose eating habits (including food choices and physical activity) can be imitated by their children. Furthermore, families are the key social context that should support healthy routines, including access to healthy food and physical activity [32, 33, 34]. Considered together, these findings suggest that parental involvement may be an important consideration in efforts to prevent and manage adolescent obesity.

Based in a collectivistic culture (such as Indonesia), peer influence is also important. Adolescents routinely seek validation within their peer networks, which can influence health behaviors for better or worse. Peers can facilitate healthy behavior change by supporting or encouraging change, responding emotionally, and engaging in activities together, such as sports participation. This result is supported by studies showing that peer‐based programs can indeed reduce body mass index in adolescents with overweight and obesity [35]. These findings imply that integrating peer networks into health promotion efforts may be a promising avenue for enhancing intervention effectiveness.

Interestingly, in this study, adolescents' motivation to manage their weight was mostly driven by appearance and less by health. This finding aligns with broader literature that demonstrates media exposure, gender norms, and societal beauty standards are significant drivers of body dissatisfaction in adolescents, particularly overweight adolescents [36, 37, 38]. An implication of these findings is that concerns about body image could potentially be considered as an entry point for future adolescent health education programs, provided that such concerns are communicated sensitively and explicitly linked to long‐term health implications.

One recognized barrier in this study is the lack of awareness among adolescents and even parents of obesity‐related health risks, including T2DM. Even among families with pre‐existing experiences of T2DM, there is inadequate healthcare professional education, insufficient public health campaigns, and inadequate health screening at the community level. Therefore, the health system needs to be more actively involved in prevention, especially through school‐ and community‐based approaches.

The findings of this study suggest the potential value of educational approaches that address body image concerns and target adolescents, parents, and peers. The aim is to raise awareness, strengthen self‐efficacy, and create supportive environments for healthy lifestyle choices. These approaches are suggested based on qualitative findings rather than being a proven intervention. Future research is needed to empirically test culturally tailored strategies that focus on family and peers can effectively promote healthy behaviors in this population, while still respecting local values [39].

## 5 Strengths and Limitations

This study has several strengths that enhance the value of its findings. First, the phenomenological design enabled an in‐depth exploration of adolescents' subjective experiences, capturing nuanced perspectives that cannot be adequately addressed through quantitative research approaches alone. Second, the study employed data triangulation by gathering information from three distinct yet interconnected participant groups (adolescents, parents, and peers), which provided a more comprehensive and multidimensional understanding of obesity dynamics within the social environment. Third, the study's cultural contextualization represents a significant strength, as it specifically captures Indonesian collectivistic values that shape health behaviors, social interactions, and family dynamics. Fourth, the use of purposive sampling ensured diversity among participants, enriching the range of perspectives captured. Fifth, the implementation of member checking and regular research team discussions throughout the data collection and analysis phases enhanced the credibility of the findings and ensured interpretative consistency.

Several limitations need to be acknowledged when interpreting the findings. First, generalizing the results is inherently limited and may not be representative of other ethnic groups or diverse geographical areas. Second, the data were collected during the COVID‐19 pandemic period from 2021 to 2022. The pandemic restrictions may have influenced adolescents' behavioral patterns and could have limited the depth of exploration and richness of non‐verbal communication. Third, the participant sample was predominantly female, indicating that the findings may more accurately reflect the experiences of adolescent girls only. Fourth, the study relied on self‐report data obtained through interviews, which are inherently prone to bias. Fifth, it is important to note that this study did not measure the effectiveness of any specific intervention program. Instead, these findings serve as a foundational contribution that can inform the development of intervention programs in the future.

## 6 Conclusions

This study contributes to the expanding literature on adolescent obesity by emphasizing the condition's complexity, particularly in collectivistic cultural contexts. The findings highlight the perceived importance of peer and family settings in shaping adolescent health habits and suggest the potential value of multi‐stakeholder, integrated, culturally sensitive approaches. Addressing appearance‐related concerns may offer a possible entry point for delivering health education while concurrently raising awareness of long‐term health risks. However, these parent and peer engagement strategies are presented as implications derived from the qualitative findings, rather than as interventions whose effectiveness has been demonstrated. Future research is needed to empirically test whether such approaches can effectively prevent T2DM and promote healthy lifestyles among adolescents.

## 7 Implications for Existing Adolescent Obesity Prevention Programs in Indonesia

- Existing Adolescent Obesity Prevention Programs in Indonesia
  Currently, several programs are implemented by the Indonesian government and health institutions, as presented in Table S1 (Existing Adolescent Obesity Prevention Programs in Indonesia). Current adolescent obesity prevention efforts in Indonesia comprise a mix of population‐level campaigns (*GERMAS* and *GENTAS*), primary healthcare capacity‐building (Motivational Interviewing training for community health providers), and school‐based interventions incorporating nutrition education and mHealth components. While these initiatives reflect commendable governmental commitment, their effectiveness is constrained by several limitations: the broad, non‐targeted nature of national campaigns may inadequately address the unique developmental and psychosocial drivers of adolescent obesity; the reach of trained healthcare providers remains limited relative to the vast adolescent population; and school‐based programs often face challenges in implementation fidelity and sustained student engagement. Collectively, these programs exhibit insufficient integration across health, education, and digital sectors, and lack robust mechanisms for long‐term behavioral reinforcement beyond the initial intervention period.
  Our findings underscore the imperative to refine these ongoing efforts by shifting from generalized knowledge dissemination toward adolescent‐centered, behaviorally anchored strategies that leverage peer influence, family involvement, and digital engagement. Given the heterogeneity in obesity risk across demographic and socioeconomic subgroups, future program revisions should prioritize: (a) co‐design with adolescents to enhance relevance and acceptability; (b) strengthening referral and follow‐up pathways between school‐based screening and primary care services; and (c) embedding rigorous process and outcome evaluations to generate context‐specific evidence on intervention effectiveness. Such adaptive, evidence‐informed enhancements are essential to translate current policy commitments into measurable and sustainable reductions in adolescent obesity prevalence across Indonesia.
- Impact of Study Findings for Program Improvement
  Based on the study findings, the following program improvements are suggested. These represent implications drawn from the qualitative data and are proposed as considerations for future program development, not as validated intervention components.
  1. Appearance‐Based Entry Point for Counseling
  Finding: Adolescents are more concerned about appearance than health.
  Suggested Program Improvement:
  ○ Health education programs may consider beginning counseling by addressing appearance concerns (lack of confidence, social interaction difficulties) as an entry point, then connect to long‐term health risks
  ○ This aligns with motivational interviewing training, showing that collaborative and participatory approaches may be more effective than direct advice‐giving.
  2 Enhanced Contextual Health Literacy
  Finding: Knowledge about obesity and T2DM risks is very low among adolescents, parents, and peers.
  Suggested Program Improvement:
  ○ Explicit T2DM risk education could be integrated into school‐based programs.
  ○ Educational materials should consider adolescents' information preferences and avoid overly medical approaches.
  ○ Parents may be involved in educational programs, as findings show low health awareness among parents as well.
  3 Strengthened Parental Engagement.
  Finding: Parents are role models for adolescents; parents' unhealthy habits are imitated by children. However, parents have great potential to support behavioral change.
  Suggested Program Improvement:
  ○ Healthcare provider training may place greater emphasis on parental involvement, as previous research showed limited changes in parental engagement aspects despite improved counseling skills.
  ○ Programs may consider educating parents about their role model function in structuring and supporting behavioral change in adolescents.
  4 Utilization of Peer Influence.
  Finding: Adolescents spend most of their time with peers, and habits (including unhealthy ones) form within friendship groups.
  Suggested Program Improvement:
  ○ School‐based programs may consider utilizing peer support and peer modeling as intervention components
  ○ mHealth application incorporating peer support features (peer feedback, joint goal‐setting) has been shown to improve engagement and intervention outcomes.
  ○ Based on our findings, group‐based interventions align with Indonesian collectivistic values and may be more effective for this population.
  5 Integration of Findings into Existing Programs.
  The findings of this study offer practical insights that can be integrated into existing health programs in Indonesia. Drawing on adolescents' perspectives regarding obesity and T2DM prevention, we identified several areas where current initiatives could be enhanced to better align with the target population's needs and social context. Table S2 (Integration of Findings into Existing Programs) summarizes the proposed additions and fine‐tuning for three key programs. First, for GERMAS/GENTAS (the national public health campaigns), we recommend incorporating an education component that explicitly links appearance and health, utilizing messages and language that resonate with adolescent audiences. Second, healthcare provider training may be refined to include modules on parental engagement and appearance‐based communication strategies in adolescent counseling. Third, school programs may benefit from embedding peer support systems as a core component, alongside organizing group physical activities that involve peers to foster social reinforcement. These proposed refinements aim to make existing interventions more contextually relevant and adolescent‐responsive, thereby enhancing their potential effectiveness in preventing T2DM among at‐risk adolescents.

## 8 Parent and Peer Engagement Processes for Intervention

Based on study findings and evidence from other studies, the following recommended parent and peer engagement processes are proposed. These processes represent implications derived from the qualitative findings and are intended as a framework for future intervention development, rather than as validated intervention protocols. Their effectiveness requires empirical testing.

- Parent Engagement Process
  Based on adolescent weight management counseling training in Indonesia, parental involvement encompasses two main roles:
  1. Structuring Role
  Drawing on our findings and prior evidence, we propose a structured framework to engage parents as active agents in adolescent obesity and T2DM prevention. Building upon the existing adolescent weight management counseling training in Indonesia, parental involvement can be operationalized through a structuring role that modifies the home environment to facilitate healthy behaviors, as detailed in Table S3 (Parents' role in supporting healthy behavior).
  Three key behavioral domains for parental structuring were food provision, physical activity, and time management. In food provision, parents are encouraged to increase the availability of vegetables, reduce fried and processed foods, and limit salt and sugar intake—aligning with our finding that adolescents ate more healthily when parents actively cooked and minimized fried foods. In physical activity, parents should invite and accompany adolescents to exercise rather than engaging in sedentary leisure, a practice that not only boosts activity levels but also models active lifestyles. In time management, parents are advised to regulate gadget use and establish consistent meal schedules, directly addressing the sedentary screen time that emerged as a significant obesity correlate in our study.
  The effectiveness of these structuring actions, however, hinges on an autonomy‐supportive communication style and must account for diverse socioeconomic and cultural contexts across Indonesian households, where access to resources and time for shared activities may vary. Future counseling training may therefore equip parents with practical strategies to overcome context‐specific barriers—such as budgeting for nutritious foods, identifying local physical activity opportunities, and collaboratively negotiating screen‐time limits with adolescents. When parents are supported with both knowledge and practical skills, they become powerful allies in sustaining behavioral changes beyond clinical or school settings.
  2 Supporting Role.
  Beyond their structuring and supervisory functions, parents also serve as critical sources of multifaceted support that can significantly influence adolescents' motivation and adherence to healthy behaviors. Table S4 (Parents in a supporting role) delineates the three primary dimensions of parental support—verbal, functional, and emotional—alongside concrete examples of how each manifests in daily interactions.
  Verbal support encompasses encouragement, advice, and motivational statements that directly reinforce health‐related behaviors. As illustrated in Table S4, verbal affirmations such as “You're overweight, it's important to exercise” represent a direct form of health communication; however, the phrasing and tone of such messages require careful consideration to avoid inducing feelings of shame or inadequacy. Effective verbal support should emphasize the positive benefits of behavior change rather than focusing solely on weight status, thereby fostering intrinsic motivation rather than external pressure.
  Functional support involves tangible actions that facilitate adolescents' access to healthy food options and exercise opportunities. This dimension bridges the gap between intention and behavior by removing practical barriers—for instance, parents who purchase nutritious groceries, prepare balanced meals, or transport adolescents to sports facilities are providing functional support that enables healthier choices. This form of support is particularly critical in contexts where adolescents lack independent mobility or financial resources to access health‐promoting resources.
  Emotional support, arguably the most delicate yet impactful dimension, centers on maintaining adolescents' positive self‐regard and psychological well‐being throughout the behavior change process. This involves avoiding negative criticism about weight or appearance, refraining from comparisons with peers, and instead offering reassurance, empathy, and acceptance regardless of the adolescent's current weight status. Research consistently demonstrates that adolescents who perceive their parents as emotionally supportive are more likely to sustain healthy behaviors and exhibit lower levels of weight‐related distress, which in turn reduces the risk of maladaptive coping patterns such as disordered eating or social withdrawal.
  Collectively, these three support dimensions are interdependent and mutually reinforcing. A parent who provides functional support (e.g., preparing healthy meals) while simultaneously delivering emotionally supportive messages and verbal encouragement creates a consistent and nurturing environment that optimizes adolescent receptivity to health interventions. Conversely, support that is perceived as controlling, critical, or conditional may undermine adolescent autonomy and precipitate resistance or disengagement. Therefore, future iterations of parent‐focused training programs should explicitly address not only what parents should do but how they should communicate and interact with adolescents in ways that preserve dignity, build self‐efficacy, and strengthen the parent‐adolescent relationship as a foundation for lifelong healthy habits.
- Peer Engagement Process
  Our finding that peer factors influence adolescents' nutritional status underscores the critical need to integrate peer‐based strategies into obesity prevention interventions. Based on these findings, Table S5 (Peer Engagement Model Based on Findings) presents a proposed peer engagement model comprising four key components: peer modeling, emotional support, peer accountability, and group activities. Peer modeling leverages adolescents' tendency to observe and emulate peers, with strategies such as training peer champions who visibly adopt healthy behaviors to normalize physical activity and healthy eating within social networks. Emotional support from close friends emerged as particularly influential, suggesting that interventions should facilitate opportunities for adolescents to build and strengthen close friendships that can buffer against social and psychological barriers to behavior change.
  Peer accountability involves mutual reminders to maintain healthy habits, shifting motivation from external authorities to the peer group itself through paired goal‐setting and reciprocal encouragement. Group activities, such as shared physical pursuits, promote both physical activity and social bonding, creating positive associations with exercise. Collectively, these four components are mutually reinforcing and should be implemented as an integrated package, enabling adolescents to internalize healthy habits as part of their social identity. This peer‐centered approach complements parental engagement, offers a scalable and low‐cost mechanism for sustaining behavior change, and may be most appropriately co‐created with adolescents to ensure cultural relevance and sustained participation. Table S5 summarizes the proposed peer engagement model, detailing the key components, implementation strategies, and representative examples from participant narratives.

## Author Contributions

**Anika Candrasari:** conceptualization, investigation, funding acquisition, writing – original draft, methodology, writing – review and editing, formal analysis, data curation, resources, validation, software, project administration, visualization. **Vina Yanti Susanti:** conceptualization, methodology, supervision, writing – review and editing. **Oryzati Hilman:** conceptualization, methodology, writing – review and editing, formal analysis, supervision. **Mora Claramita:** conceptualization, writing – review and editing, validation, formal analysis, supervision, visualization, data curation.

## Funding

This work was supported by the Indonesia Endowment Fund for Education (LPDP) Ministry of Finance under Grant (KET‐969/LPDP.4/2020).

## Ethics Statement

The Medical and Health Research Ethics Committee of Universitas Gadjah Mada granted ethical permission for this study (Reference No. KE/FK/1382/EC/2020).

## Consent

Informed consent was obtained from all subjects involved in the study.

## Conflicts of Interest

The authors declare no conflicts of interest.

## Supporting information


**Table S1:** Existing adolescent obesity prevention programs in Indonesia.
**Table S2:** Integration of findings into existing programs.
**Table S3:** Parents' role in supporting healthy behavior.
**Table S4:** Parents in a supporting role.
**Table S5:** Peer engagement model based on findings.

## Data Availability

A summary of the data that support the findings of this study is available on request from the corresponding author, AC. The data are not publicly available owing to their containing information that could compromise the privacy of research participants.
