# Supplementary material for: Appearance Over Health: A Phenomenological Study of Obese Adolescents With a Family History of Type 2 Diabetes Mellitus in a Collectivistic Indonesian Context
Source: J Gen Fam Med. 2026 Oct 22;27(6):e70190. doi: 10.1002/jgf2.70190 (PMC13690096; doi:10.1002/jgf2.70190)
Supplement: Supplementary file 1 [file JGF2-27-e70190-s001.docx]

**Table S1.** Existing Adolescent Obesity Prevention Programs in Indonesia

| Program | Description | Target |
| --- | --- | --- |
| *GERMAS* (Healthy Living Community Movement) | Focuses on regular physical activity and healthy lifestyles | General population, including adolescents |
| *GENTAS* (Archipelago Movement to Reduce Obesity Rates) | Obesity management through nutrition regulation and physical activity enhancement | General population |
| Adolescent Weight Management Counseling Training | 4-week online training for primary healthcare providers using Motivational Interviewing | Primary care physicians and nurses at community health centers |
| School-Based Programs | Nutrition education and health promotion in schools, including mHealth-based interventions | School-aged adolescents |

**Table S2.** Integration of Findings into Existing Programs

| Program | Addition/Fine Tuning |
| --- | --- |
| GERMAS/GENTAS | Add an education component explicitly linking appearance and health; use messages relevant to adolescents |
| Healthcare Provider Training | Enhance modules on parental engagement and appearance-based approaches in adolescent counseling |
| School Programs | Incorporate peer support systems as a core component; organize group physical activities involving peers |

**Table S3.** Parents' role in supporting healthy behaviour

| Aspect | Recommended Action | Based on Finding |
| --- | --- | --- |
| Food Provision | Provide vegetables, reduce fried foods, limit salt/sugar | "Parents get involved in cooking, and reduce fried food" |
| Physical Activity | Invite and accompany adolescents to exercise | "Invite sports, rather than sitting or relaxing" |
| Time Management | Limit gadget use, regulate meal schedules | Related to sedentary behavior |

**Table S4.** Parents in a supporting role

| Support Aspect | Implementation |
| --- | --- |
| Verbal Support | Encouragement, advice, motivation ("You're overweight, it's important to exercise") |
| Functional Support | Facilitating access to healthy food and exercise facilities |
| Emotional Support | Maintaining adolescents' positive feelings, avoiding negative criticism about weight |

**Table S5**. Peer Engagement Model Based on Findings

| Component | Implementation | Example from Findings |
| --- | --- | --- |
| Peer Modeling | Adolescents observe peers exhibiting healthy behaviors | "Friends invite to exercise together" |
| Emotional Support | Emotional support from close friends | "Encouraging, especially if we're close friends" |
| Peer Accountability | Mutual reminders to eat healthily and exercise | "I want friends also to invite me to do sports" |
| Group Activities | Shared physical activities | "We often went up the mountain" |
